# Psychometric analysis of Anatomy MCQs in Modular examination

**DOI:** 10.12669/pjms.335.12382

**Published:** 2017

**Authors:** Zia ul Islam, Ambreen Usmani

**Affiliations:** 1 Prof. Dr. Zia ul Islam, M.Phil. Prof. and Head, Department of Anatomy, Liaquat National Hospital and Medical College, Karachi, Pakistan; 2 Prof. Dr. Ambreen Usmani, M.Phil, MCPS (HPE), PGD Bioethics, PhD- Anatomy. Prof. and Head, Department of Anatomy, Bahria University Medical and Dental College Karachi, Pakistan

**Keywords:** Difficulty index, Discriminatory index, Modules, Reliability, MCQ, Anatomy

## Abstract

**Objective:**

To analyze the psychometric indices of Anatomy question items in modular system assessment.

**Methods:**

A quantitative study was done to determine the quality of MCQs and to analyze the performance of 1^st^ year 100 MBBS students. Each module covers different subjects of MBBS curriculum but psychometric analysis was done on the subject of Anatomy only. The assessment results of 3 modules were taken and checked by item analysis to see the mean differences between the modules using ANOVA. Post hoc analysis was determined by using Tukey HSD test.

**Results:**

A total of 140 one best (OB) Anatomy MCQ items were calculated for difficulty index, discriminatory index and reliability. Difficulty index was found to be higher in module I when compared with module II and III. Discriminatory index comparatively showed higher results in module II whereas reliability of module III was significantly higher than the other modules. Results were considered to be significant with p value≤ 0.05.

**Conclusions:**

The psychometric analysis of Anatomy MCQs showed average difficulty, good discrimination and reliability.

## INTRODUCTION

Assessment plays a very important role in education at all stages of learning from school to Universities. If assessments are properly planned and implemented then they produce a very powerful effect on learning and curriculum. A well-made assessment tool that tests objectives relevant to the course is an important characteristic of an exam. The course objectives must be given as a guide to the students and the outcome of educational program must be clearly defined.^1^

One best (OB) Multiple choice question (MCQ) is a common tool used to assess the cognitive capability of the student.^2^ MCQs have certain advantages like feasibility and objectivity, this tool can assess several concepts depending upon the number of questions in a relatively short time. MCQs if constructed properly, may test higher levels of cognitive reasoning and discriminate between students who are high and low achievers.^2^,^3^ The MCQ format has some limitations like good quality MCQ are relatively difficult but not impossible to construct for example problem solving questions. It has been observed that few teaching faculty have adequate education and training in developing standard quality OB MCQs.^2^ An effective OB MCQ can be described in terms of overall item, the stem and the options.^4^ Items writing flaws are the technical errors that can be present in any MCQ and they can affect student’s performance. MCQ`s which are poorly constructed on the basis of item writing flaws like unclear or unfocused stems, negatively worded options, options which are not plausible or contains irrelevant information affects the quality of the MCQs^5^ and makes the assessment process less reliable & valid.^2^,^3^

In medical colleges, Anatomy OB MCQs are considerably based on recall or factual knowledge and some of these MCQs have item flaws which gives a take home message that rote learning is required only, but if the questions are related to relevant clinical scenarios in which Anatomy can be linked to, than the students will take more interest as they will be able to understand the relevancy of this vast subject.^4^ Literature shows that about 40–45% of the MCQ questions contained violation of item writing guide lines and over 90% were written at low cognitive levels.^2^,^4^ No empirical data is available regarding MCQ item analysis in the subject of Anatomy in our medical colleges.^5^ The assessment of learning in several universities is based on comparison of educational performance with educational purposes and expectations.^6^ Teaching institutes should make an effort to identify and resolve all short comings to facilitate active and constructive learning.^7^ In this regard, this research has conducted psychometric analysis of OB Anatomy MCQs to improve the quality of assessment. Therefore the aim of this study was to analyze the psychometric indices of items in OB Anatomy MCQs assessment tool given in modules.

## METHODS

This study was conducted in the Department of Anatomy at Bahria University Medical and Dental College as a part of their MBBS module assessment in the subject of Anatomy. Permission was obtained from ethical review committee of the Institute to carry out this research. The number of students were 143 studying in 1^st^ year MBBS and they were clearly explained about the study and an informed consent form was filled by each student to seek their permission to use their results but they were assured that their names will not be mentioned.

Out of 143 students, one hundred first year MBBS students voluntarily opted to take part in this study. Written informed consent forms were given to the students before each module, the students were informed that their names will be given a code when entering the data for statistical analysis. Only results of those students were mentioned who consented to be a part of the study. The same students were inducted for all 3 modules.

Module-I consist of cell biology and genetics. The second module consists of Musculoskeletal/blood and lymphoid tissue. While the third module consist of Cardiovascular and Respiratory system. One hundred and forty items (in module papers) comprising with four options and one correct without any negative marking are selected from these modular exams. These items are constructed to assess various levels of knowledge according to bloom’s taxonomy.^8^

These 140 items are selected from the existing MCQ bank after a pre-validation by the MCQ review committee of the department. All the modular examinations are in pencil and paper format.^9^ Test results were assessed by Optical Machine Reader with a are computed using custom software program.

Post validation of the MCQ’s regarding each module was done independently and a comparison was made between the three modules in context to difficulty index, discrimination index and reliability across these modular exams. On the basis of these indices we analyze the standard of each modular exam along with the present status of the MCQs bank and its future up gradation.

### Data Analysis

Each item in every modular exam was analyzed for the following

***1. Difficulty index or facility value or “P” value***.

It was calculated by using the formula.

p= H+L / Nx100

Where; **H=** Number of students answering the items correctly in the high achieving group.

**L =** Number of students answering the items correctly in Low achieving group.

**N=** Total number of students in the two groups (including non-responders).

Difficulty index between 30-70% are considered as acceptable. Those items with values between 50% to 60% are ideal while items with less than 30% (too difficult) and more than 70% (too easy) are not acceptable or need revision.

***2. Discrimination index(DI)*** or (Point Biserial) was calculated using the formula

D= H – L X 2 / N

Where the symbols **H, L** and **N** represent the same values as mentioned above.

The discrimination index is a statistic that indicates the extent to which an item has discriminated between the high achievers and low achievers on the test. The index is represented as a fraction and varies between (- 1 to 1) optimally an item should have a positive discrimination index of at least 0.2, which indicates that high scorers have a high probability of answering correctly and low scorers have a low probability of answering correctly. Items with negative indices should be examined to determine whether the items is flawed or mis-keyed.^9^

***3. Reliability Coefficient (ALPHA)***.

It is a measure of the amount of measurement error associated with a test score. This is being calculated by using KR20 formula. The range varied from 0.0 to 1.0. The higher the value, the more reliable the overall test scores. The reliability coefficient indicated how well the items are correlated with one another. Higher reliability also indicates that all items are measuring the same thing. By using this parameter each module exam will reflect the internal consistency of the items.^10^

### Statistical Analysis

Data was stored and analyzed using SPSS 16, mean and standard deviation were reported for Difficulty index, Discrimination and Reliability measures of three module exams with total 140 items, 95% confidence interval for mean and p-value were also reported to see the mean differences of these measures across modules using one way analysis of variance (ANOVA). Post hoc analysis using Tukey HSD test was done for those measure that were gives significant results in ANOVA, reliability of the data was checked using Cronbach alpha. All p-values less than 0.05 were considered statistically significant.

Count and percentages were reported for the difficulty index, discrimination and reliability outcomes after categorizing into standard given levels. Multiple bar chart and pie chart were also used to give the graphical presentation of study outcomes.

## RESULTS

**Fig.1:**
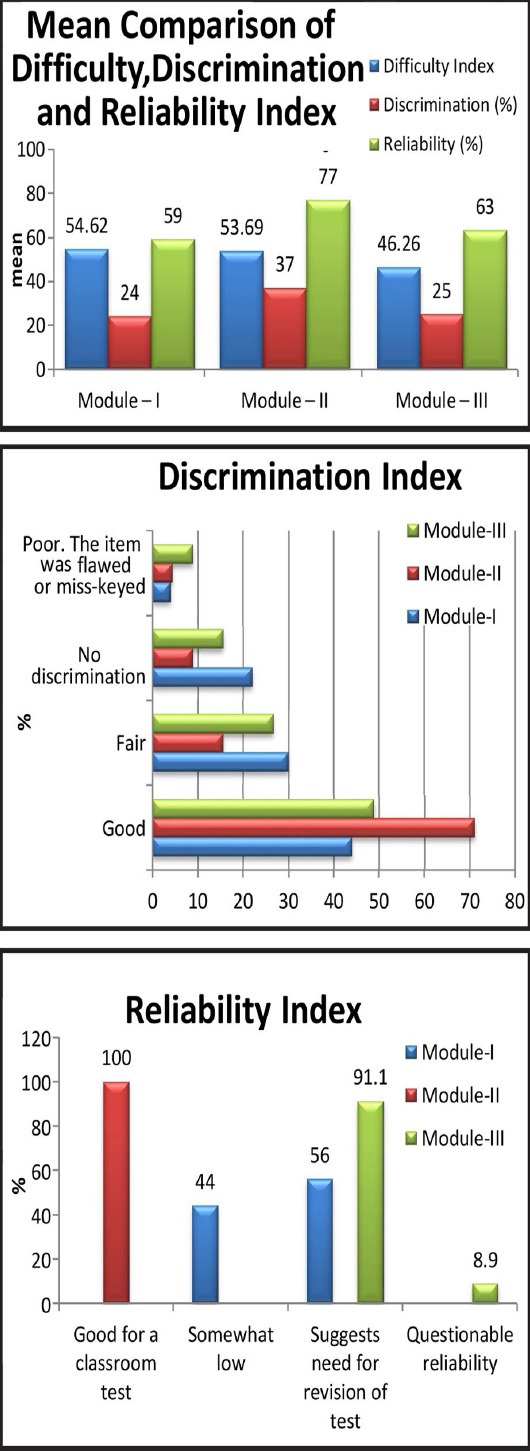
Analysis of All Three Modules.

Table-I compares the mean of Difficulty index, Discrimination and Reliability across three modules exams of students; results showed that the mean difficulty index in module-I was higher as compared to next two modules exams, in Module-II and Module-III. (Table-I). However, using one way ANOVA did not give any evidence of statistical significant mean differences in difficulty index across three modules exams with p-value 0.173. (Fig.1)

**Table-I:** Mean Comparison of Difficulty index, Discrimination and Reliability across three Modules.

*Measures*		*Mean*	*Standard Deviation*	*95% Confidence interval for mean*		*p-value*

				*Lower Bound*	*Upper Bound*	
Difficulty Index	Module–I (N=50 items)	54.62	26.16	47.19	62.06	0.173
	Module–II (N=45 items)	53.69	20.82	47.43	59.95	
	Module–III (N=45 items)	46.26	22.35	39.55	52.98	
	Total	51.63	23.46	47.71	55.56	
Discrimination	Module–I (N=50 items)	0.24	0.18	0.19	0.29	<0.01*
	Module–II (N=45 items)	0.37**	0.20	0.30	0.43	
	Module–III (N=45 items)	0.25	0.21	0.18	0.31	
	Total	0.28	0.20	0.25	0.32	
Reliability	Module–I (N=50 items)	0.59***	0.01	0.59	0.59	<0.01*
	Module–II (N=45 items)	0.77***	0.005	0.77	0.77	
	Module–III (N=45 items)	0.51***	0.016	0.51	0.52	
	Total	0.63***	0.10	0.61	0.64	

* p<0.05 was considered Significant using one way ANOVA.

** Module II results found significantly different from Module I and Module III using Tukey HSD test.

*** All three module results are significantly different with each other Tukey HSD test.

Discrimination in module-II was higher as compare to Module-I and Module- III exam results, and ANOVA test with p-value less the 0.01 gives the evidence that mean discrimination across three modules was not same, for further investigation post-hoc analysis was done using Tukey HSD test which revealed that Module –II discrimination index was significantly different from Module-I and Module-III, whereas there was no significant differences found for discrimination between Module-I and Module-III exams.(Fig.2)

Reliability of module-II exam was significantly higher than Module-I and Module-III exams, ANOVA results showed that all there modules exams have significant differences for the reliability with p-value less than 0.01(Table-II).

**Table-II:** Comparison of Measures outcome across three Modules.

*Measures Outcome*		*Module*					

		*I (n= 50 items)*		*II (n=45 items)*		*III (n= 45 items)*	

		*n*	*%*	*n*	*%*	*n*	*%*
Difficulty Index	Easy	15	30	6	13.3	5	11.1
	Average	30	60	34	75.6	32	71.1
	Hard	5	10	5	11.1	8	17.8
Discrimination	Good	22	44	32	71.1	22	48.9
	Fair	15	30	7	15.6	12	26.7
	No discrimination	11	22	4	8.9	7	15.6
	Poor: The item was flawed or miss-keyed	2	4.0	2	4.4	4	8.9
Reliability	
	Good for a classroom test	-	-	45	100.0	-	-
	Somewhat low	22	44.0	-	-	-	-
	Suggests need for revision of test	28	56.0	-	-	41	91.1
	Questionable reliability	-	-	-	-	4	8.9

Table-II gives the categorical analysis on difficulty index, Discrimination and Reliability analysis of three modules exams outcomes, it was found that, in Module-I out of 50 items, 60% were found average with difficulty index from 26%-74%, in Module-II exams this was increased and found 75.6% out of 45 items, similarly in Module-III exam it was 71.1% out of 45 items, the highest percentage of difficulty index for Hard category found in Module-III exam, which was 17.8% with difficulty index 25% or below.

For discrimination 44% items of Module-I exam found as “Good” with range of Discrimination from 0.30 and above, in module-II exam it was 71.1% and in Module-II exam it was lowest 48.9% in Module –III exam 8.9% items were found “poor” or miss keyed, with Negative discrimination, In Module –I exam it was 4% only and in Module-II exam it was 4.4%.

Results of Reliability analysis showed that in all three modules there were none of the item as Excellent with reliability 0.90 or above, or very good reliability with reliability index from 0.80-0.90, however, in Module-II exams all 45 items were found Good, with reliability from 0.60-0.70 in module–III exams 91.1% items were found with revision of test the reliability was 0.50 – 0.60, and 8.9% items were found with questionable reliability with index 0.50 or below. Results of reliability analysis using Cronbach alpha showed that, difficulty index had weak reliability, while Discrimination and Reliability index were showed significance. (Fig.1) Tukey’s test were used to compare each question among the 3 modules of the students.

## DISCUSSION

Competency assessment is the strength to educate medical students for being tomorrow’s doctors. Assessment not only shapes students but also reflects their learning approaches and performances.^11^,^12^ The best assessment plays a vital role to offer insight in students’ clinical abilities and overall achievement.^13^,^14^ The assessment tools are thus aligned to modify cognitive abilities of students to achieve maximum efficiency which would help them to identify problems, solve them, think critically and interpret findings measure up to this level rather than just assessing recall and factual knowledge.^15^ Inappropriate design of assessment formats may lead to unwanted outcomes of competencies and types of patient care.^14^

In the summative examinations, it is expected that level of difficulty for a four-option multiple choice test should be between 60% and 80%. The mean difficulty index of all the modules in our study was 46% to 54%. Our study shows similar results to other studies one of which observed 48% item difficulty level in examination of items from subject of basic medical sciences for the licensure of registered nurses examination conducted in Taiwan.^16^ The difficulty index from 47.17 ± 19.79 to58.8 ± 19.33was documented by Karelia et al.^17^ On the contrary, item analysis of MCQ done by Si Mui Sim et al. revealed that 40% of MCQ`s in the assessment crossed the difficulty index of 70% making the test items easy for the students.^18^ Other study by Patel and Mahajan showed 80% of items in the acceptable range which can be compared to our study.^19^

Properly constructed multiple choice questions assess higher-order cognitive processing of Bloom’s taxonomy such as interpretation, synthesis and application of knowledge, instead of just testing recall of isolated facts.^20^,^21^ It is recommended that an ideal item (MCQ) is the one which has average difficulty (between 31 and 60%), high discrimination (DI ≥ 0.25) and maximum DE (100%) with three functional distractors.^22^ Moderately easy/difficult items had the maximal discriminative ability. Sometime very difficult item display poor discrimination, but the very easy item had high discrimination index, indicating a faulty item, or incorrect keys. The discrimination index in our study was 28% in all the modules which is comparable to Khan HF et al who observed discrimination index of more than 20% in the MCQs of all summative tests.^21^

In Module III exam maximum (8.9%) items were found “poor” with negative discrimination in comparison to Module I and II exam. Research shows that due to miss keyed answers in which the key is wrong the poorer items are calculated. Therefore, it must be mandatory to review and re-check the answers. This can be described on the basis of defect in the construction, difficult concepts relative to the overall ability of the class, indefinite or not clear statements or may be more than one correct option. As far as the reliability is concerned, module-II exam had maximum reliability from 0.60 – 0.70. Reliability is basically concerned with the ability of an instrument to measure constantly. It also shows the amount of measurement error in a test.^23^ Our research is in accordance with Tavakol M et al. which shows that module II items are more correlated to each other because of high reliability index as compared to module I & especially module III items which shows a less reliability.^24^ It has been seen in many cases that high coefficient alpha does not always means a high degree of internal consistency. Alpha is also affected by low number of items, interrelatedness of items and dimensionality.^24^

Our study has limitations that the number of items are not greater in numbers in each module and number of good quality items in MCQ bank are less. This is the first study done in the subject of Anatomy that can bring a change in the construction and selection of OB MCQ`s in Anatomy examination for proper assessment approach in curriculum development. Study thus emphasizes the need for selection of quality OB MCQs which truly assess the knowledge and are able to differentiate the students of different abilities in correct manner in each module for all disciplines.

## CONCLUSION

The item analysis of Anatomy MCQs in three modules at BUMDC displayed criteria of average difficulty and good discrimination and reliability in an item in all the three modules which helped high achievers to obtain high scorers. The better results in module II need to be further explained in terms of either the selection criteria or any other factor that accounted for higher reliability results.

### Recommendations

It is recommended through this research that post hoc analysis of items is a very important tool to identify the deficiencies and weakness in the construction of good quality MCQ`s. In this way, the question bank can have a good number of reliable and valid pool of MCQ`s resulting in more authentic assessment in the subject of anatomy.
